# The clinical outcome of emergency superficial temporal artery-to-middle cerebral artery bypass in acute ischemic stroke with large vessel occlusion

**DOI:** 10.1007/s10143-023-02257-0

**Published:** 2024-01-02

**Authors:** Lei Guo, Li Yang, Chunling Li, Yi Zeng, Ruxiang Xu, Zhengyu Wang, Chonggui Jiang

**Affiliations:** Department of Neurosurgery, Sichuan Academy of Medical Sciences and Sichuan Provincial People’s Hospital, University of Electronic Science and Technology of China, Chengdu, 610072 China

**Keywords:** Superficial temporal artery-to-middle cerebral artery bypass, Acute ischemic stroke, Large vessel occlusion, Cohort study, Meta-analysis

## Abstract

The role of superficial temporal artery-to-middle cerebral artery (STA-MCA) bypass in acute ischemic stroke (AIS) is contentious, with no evidence in patients with AIS and large vessel occlusion (AIS-LVO). We conducted a cohort study to assess emergency STA-MCA outcomes in AIS-LVO and a meta-analysis to evaluate STA-MCA outcomes in early AIS treatment. From January 2018 to March 2021, we consecutively recruited newly diagnosed AIS-LVO patients, dividing them into STA-MCA and non-STA-MCA groups. To evaluate the neurological status and outcomes, we employed the National Institutes of Health Stroke Scale (NIHSS) during the acute phase and the modified Rankin Scale (mRS) during the follow-up period. Additionally, we conducted a meta-analysis encompassing all available clinical studies to assess the impact of STA-MCA on patients with AIS. In the cohort study (56 patients), we observed more significant neurological improvement in the STA-MCA group at two weeks (p = 0.030). However, there was no difference in the clinical outcomes between the two groups. Multivariable logistic regression identified the NIHSS at two weeks (OR: 0.840; 95% CI: 0.754–0.936, p = 0.002) as the most critical predictor of a good outcome. Our meta-analysis of seven studies indicated a 67% rate for achieving a good outcome (mRS < 3) at follow-up points (95% CI: 57%–77%, I^2^ = 44.1%). In summary, while the meta-analysis suggested the potential role of STA-MCA bypass in mild to moderate AIS, our single-center cohort study indicated that STA-MCA bypass does not seem to improve the prognosis of patients who suffer from AIS-LVO.

**Supplementary Information:**

The online version contains supplementary material available at 10.1007/s10143-023-02257-0.

## Introduction

Acute ischemic stroke (AIS) resulting from large vessel occlusion (LVO) is a highly life-threatening condition, carrying a more than twofold increased risk of death and permanent disability compared to non-LVO-related ischemic stroke [1]. Over the past decade, numerous randomized clinical trials have showcased the transformative potential of endovascular thrombectomy (EVT) in AIS-LVO treatment [2–7]. Despite the recognized clinical advantages of EVT, challenges related to time constraints and the complexity of vascular lesions limit its applicability [8, 9]. Approximately 3.3%–12.9% [10–12] of EVT procedures encounter obstacles such as guide catheter failure or post-EVT reocclusion. The possibility of suboptimal outcomes, such as incomplete restoration of hemodynamics [13], is also a stark reality. Therefore, future directions in AIS-LVO treatment should prioritize precision medicine, interdisciplinary collaboration, technological advancements, expanded treatment timeframes, and improved treatment accessibility.

Superficial temporal artery-to-middle cerebral artery (STA-MCA) bypass surgery is typically recognized for its role in preventing brain infarction and reducing hemorrhage risk by enhancing cerebral hemodynamics in patients with steno-occlusive cerebrovascular diseases [14]. Recent studies have indicated that urgent STA-MCA bypass can reduce infarct size, enhance neurological function, improve quality of life, and lower mortality in AIS patients who are ineligible for or have failed EVT [15, 16]. However, none of these studies have specifically addressed the impact of urgent STA-MCA bypass in resolving AIS-LVO in patients with a relatively high National Institutes of Health Stroke Scale (NIHSS) score during the acute phase. To further evaluate its effectiveness and safety in this subgroup, we conducted a cohort study to assess the clinical outcomes of emergency STA-MCA in AIS-LVO and a meta-analysis to evaluate the clinical outcomes of STA-MCA performed in the early stages of AIS treatment.

## Materials and methods

### Cohort study

#### Patients

From January 2018 to March 2021, we consecutively enrolled newly diagnosed AIS-LVO patients in our department. We categorized the patient population into two groups: the STA-MCA group and the non-STA-MCA group. An emergency STA-MCA bypass was defined as a procedure that was performed within less than seven days from symptom onset. Inclusion criteria for the STA-MCA group were: (1) age > 18 years; (2) confirmed anterior circulation AIS-LVO via digital subtraction angiography (DSA) or computed tomography angiography (CTA); (3) perfusion-diffusion mismatch (PDM) or symptom diffusion mismatch (mismatch between clinical severity and infarction); (4) reocclusion after recanalization with thrombolysis or EVT; and (5) neurological deterioration > two-point decrease in NIHSS scores despite maximal medical treatment. Patients meeting these criteria but declining STA-MCA bypass during the same period were included in the non-STA-MCA group. This study was ethically reviewed by our institution's ethics committee, and all human data in the study adhered to the Declaration of Helsinki. Informed consent forms were legally signed by all patients and their representatives.

#### STA-MCA surgery

**Fig. 1:**
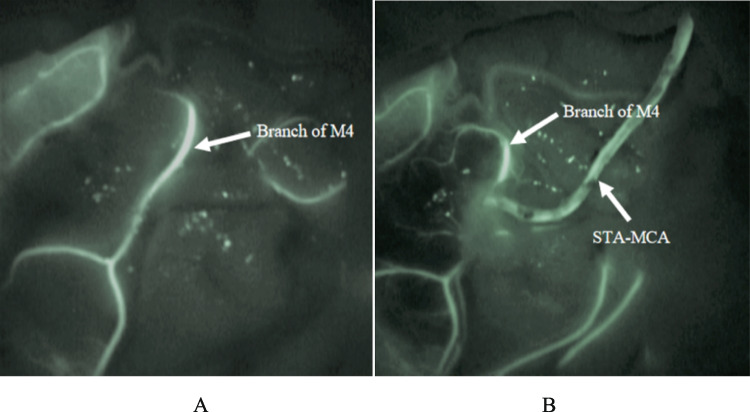
Intraoperative ICG angiograms before (**A**) and after (**B**) anastomosis

We utilized a handheld Doppler ultrasound to chart the STA's course, and the parietal or frontal STA branch was dissected and used as the donor artery. The surgeon selected one of the M4 branches in the area with perfusion deficits as the recipient vessel. Subsequently, end-to-side anastomosis between the donor and recipient vessels was performed. This anastomosis was routinely executed using continuous running a 10–0 Prolene suture, and intraoperative assessment of bypass patency was conducted via indocyanine green (ICG) video angiography and micro-Doppler (Fig. 1A and B).

#### Management

We collected clinical data, which included patient demographics, follow-up duration, medical history, blood routine examination results, details of medical treatments (intravenous recombinant tissue plasminogen activator and EVT), the lesioned artery, time from onset/aggravation to surgery (deterioration time), and the use of decompressive craniectomy (DC).

All patients were continuously monitored for a minimum of seven days in the neurosurgical intensive care unit. After STA-MCA bypass surgery, meticulous blood pressure control was maintained, with a target range set slightly below normal, aiming for systolic blood pressure between 120 and 130 mmHg. In the early postoperative period, patients in the STA-MCA group underwent CTA or transcranial Doppler (TCD) examinations to assess the patency of the anastomoses.

#### Outcomes and follow-up

After hospital discharge, patients received monthly follow-up assessments for at least one year at the neurosurgery clinic. Neurological status was assessed by the NIHSS score, and post-surgery assessments included the modified Rankin Scale (mRS) score. The NIHSS score was assessed upon admission and at two weeks post-onset, while the mRS score was measured at three months and one year. The primary outcome, evaluated at one year post-treatment, was defined as a good outcome when the mRS score was 0–2. Secondary outcome measures included assessing hemorrhagic transformation (HT), recurrent ischemic stroke, all-cause mortality, and stroke-related mortality.

### Statistical methods

All statistical analyses were performed using GraphPad Prsim 8.0 (GraphPad Software, Inc., USA). Continuous variables were reported as mean ± standard deviation (SD) or median with interquartile range (IQR), and categorical variables were reported as the number with percentage. Continuous variables were compared between the two groups using the Mann–Whitney U test or Student’s t test, and categorical variables were compared using χ 2 or Fisher’s exact test (the expected frequency of events < 5) as appropriate. Univariate and multivariate logistic regression were used to assess the independent factors associated with a good outcome. A two-sided P < 0.05 was considered statistically significant.

### Meta-analysis

#### Search strategy and inclusion criteria

Following the Preferred Reporting Items for Systematic Reviews and Meta-Analysis (PRISMA) guidelines (Supplementary Table 1), we conducted this meta-analysis. The review protocol was registered with PROSPERO (CRD42022320907). Our inclusion criteria encompassed trials that were (1) randomized controlled trials (RCTs) or clinical studies reporting on STA-MCA bypass for AIS, and (2) published as original articles in English. We excluded studies designed as nonhuman investigations or case reports, those lacking primary data analyses (e.g., letters, editorials, or narrative reviews), and studies that did not provide sufficient data. A full-text screening process was carried out to select pertinent articles for data extraction.

#### Data extraction and quality assessment

Two independent reviewers (Li Yang and Da Huang) systematically searched multiple databases, including PubMed/Medline, Embase, Web of Science, and the Cochrane Library, from their inception until January 29, 2023. Our search terms encompassed MeSH headings and keywords related to "Superficial Temporal Artery to Middle Cerebral Artery Bypass Surgery" and the MeSH heading "Acute Ischemic Stroke." Additionally, we explored ClinicalTrials.gov for ongoing or unpublished eligible studies.

Using a standard form, the same two reviewers (Li Yang and Da Huang) independently extracted data from the selected studies. This data included information such as the first author, publication year, country of origin, sample size (comprising both the number of cases and total participants), baseline characteristics of patients enrolled in clinical trials, and clinical outcomes of STA-MCA patients. The methodological quality of the included studies was assessed using the Methodological Index for Non-Randomized Studies (MINORS), which consists of 12 methodological points. The first eight points are applicable to both noncomparative and comparative studies, while the remaining four are specific to studies with two or more groups [17].

### Statistical analysis of the meta-analysis

We conducted all statistical analyses using Stata software version 16.0 (Stata Corporation, College Station, Texas). Our primary outcome measure was the incidence of a good outcome, defined as an mRS score of 0–2 at the last follow-up in patients treated with STA-MCA for AIS. To assess heterogeneity among eligible studies, we employed the Q test [18] and the I^2^ score [19]. Given the observed heterogeneity, we used random-effects models.

We opted not to assess publication bias through Begg's funnel plot due to the limited number of included studies [20]. Instead, we conducted Egger's test to evaluate the potential for publication bias, with a P-value less than 0.05 indicating the presence of small-study effects. Additionally, we performed sensitivity analysis to assess the robustness of our primary efficacy indicators.

## Results

### Cohort study

#### Clinical characteristics of patients

We included 63 patients aged 18 years or older with newly diagnosed AIS-LVO in this study, of whom 29 underwent STA-MCA, with 7 of them showing failed blood flow by CTA or TCD within three days after surgery. A total of 56 patients (34 in the non-STA-MCA group and 22 in the STA-MCA group) with a mean age of 61.29 years were enrolled and completed follow-up. Patient characteristics are summarized in Table 1. No significant differences were observed between the two groups, except for the NIHSS score at two weeks after surgery.

**Table 1:** Clinical characteristics of the STA-MCA and control groups

Characteristic	All (*n* = 56)	Non-STA-MCA (*n* = 34)	STA-MCA (*n* = 22)	t/χ 2/U	*P*
Age, years	61.29 ± 11.52	63.94 ± 11.06	58.64 ± 11.98	1.697	0.096
Sex/Male	31 (55.4%)	19 (55.9%)	12 (54.5%)	0.01	0.098
Weight, Kg	63.83 ± 10.33	64.56 ± 12.14	63.09 ± 8.51	0.493	0.624
Follow-up time, months	12.11 ± 4.30	12.44 ± 4.78	11.35 ± 2.50	0.953	0.345
Risk factors, *n*(%)					
Hypertension	28 (50.0%)	20 (58.8%)	8 (36.4%)	2.7	0.100
Diabetes mellitus	9 (16.1%)	7 (8.8%)	2 (9.1%)	Fisher	0.458
Hyperlipemia	3 (5.4%)	1 (2.9%)	2 (9.1%)	Fisher	0.555
Smoking	11 (19.6%)	6 (17.6%)	5 (22.7%)	Fisher	0.736
Alcohol	10 (17.9%)	6 (17.6%)	4 (18.2%)	Fisher	0.999
Coronary heart disease	11 (19.7%)	8 (23.5%)	3 (13.6%)	Fisher	0.498
Atrial fibrillation	14 (25.0%)	11 (32.4%)	3 (13.6%)	Fisher	0.205
Laboratory data					
WBC,10^9/L	10.57 ± 4.03	10.69 ± 3.34	10.44 ± 4.824	-0.2	0.843
Hb, g/L	122.02 ± 26.36	120.13 ± 27.85	124.30 ± 24.97	0.52	0.607
Plt, 10^9/L	180.52 ± 60.44	174.75 ± 66.45	187.45 ± 53.19	0.69	0.49
hsCRP, mg/L	35.53 (3.28–97.47)	51.25(2.90–98.51)	11.43(3.83–97.47)	419	0.465
Alb,g/L	32.40 (29.63–97.47)	32.10(30.43–40.10)	33.20(27.88–39)	434	0.706
osmoticpressure, mOsm/L	288.0 (278.0–299.0)	289.0(287.25–300.5)	279.0(274.00–298.75)	379	0.094
Treatment, *n*(%)					
IV rt-PA	13 (23.1%)	10 (29.4%)	3 (13.66%)	Fisher	0.210
EVT	45 (80.4%)	29(85.3%)	16 (72.7%)	0.29	0.584
Lesion, *n*(%)					
ICA	18 (32.1%)	10 (29.4%)	8 (36.4%)	0.3	0.586
MCA	38 (67.9%)	22 (64.7%)	16 (72.7%)	0.716	0.397
Surgery-related data					
Infarct core, ml	104(89–115)	103(86–116)	108(99–116)	313	0.311
Penumbra, ml	157(140–189)	153(133–189)	169(141–192)	298	0.205
Penumbra/infarct core ratio	1.49(1.30–1.74)	1.43(1.29–1.76)	1.62(1.41–1.72)	307.5	0.269
Deterioration time, hours	19.25 (26.75–48.375)	25.6(19–35.8)	33(20–52.375)	497	0.311
DC, *n*(%)	26 (46.4%)	17 (50.0%)	9 (40.9%)	0.438	0.505
Neurological score					
Initial NIHSS	15.00 (12.25–25.00)	15.00 (12.75–20.25)	16.00 (12.00–26.75)	318.5	0.355
NIHSS at 2 weeks	20.50 (15.00–35.00)	26.50 (15.75–35.00)	18.00 (8.00–35.00)	246	0.030*
3 month-mRS	4.00 (3.00–6.00)	5.50 (3.00–6.00)	3.50 (3.00–6.00)	284.5	0.114
1 year-mRS	5.00 (3.00–6.00)	5.50 (3.00–6.00)	4.00 (3.00–6.00)	288	0.132

*STA-MCA* superficial temporal artery-middle cerebral artery, *WBC* white blood cell, *Hb* hemoglobin, *Plt* platelet, *hsCRP* hypersensitive C-reactive protein, *Alb* albumin, *IV* intravenous, *rt-PA* recombinant tissue plasminogen activator, *EVT* endovascular treatment, *ICA* internal carotid artery, *MCA* middle cerebral artery, *Deterioration time* time from onset/aggravation to surgery, *DC* decompressive craniectomy, *NIHSS* National Institutes of Health Stroke Scale, *Initial NIHSS* NIHSS at admission, *NIHSS at 2 weeks* NIHSS score at 2 weeks after onset, *mRS* modified Rankin Scale; * *p* < 0.05

Neurological status was assessed using the NIHSS during the acute phase. In the STA-MCA group, the median initial NIHSS score was 15 (IQR: 12.75–25.25), while the median preoperative NIHSS score was 35 (IQR: 20–35). This decreased to 26.50 (IQR: 15.75–35.00) at two weeks after onset. The STA-MCA group showed more pronounced improvement in neurological status (NIHSS at two weeks: 18 [IQR: 8–35] vs. 26.50 [IQR: 15.75–35], p = 0.030). At three months after onset, the median mRS score was 4 (IQR: 3–6), and the median 1-year mRS score was 5 (IQR: 3–6). There were no significant differences in mRS scores between the two groups.

#### Follow-up and outcomes

During the follow-up period (mean: 12.11 ± 4.30 months), 19 patients (25.0%) achieved good outcomes based on their 1-year mRS scores. Intracranial hemorrhage occurred in 23 patients (41.1%), while 13 patients (23.2%) experienced recurrent stroke. The all-cause mortality rate was 23.2%, and stroke-related mortality was 39.3%. There were no significant differences in clinical outcomes between the two groups (Table 2).

**Table 2:** Clinical outcome of patients

Outcome	All (*n* = 56)	Non-STA-MCA (*n* = 34)	STA-MCA (*n* = 22)	χ 2	*P*-value
Good Outcome, *n* (%)	19 (33.9%)	11 (32.4%)	8 (36.4%)	0.096	0.757
Hemorrhagic transformation, *n* (%)	23 (41.1%)	12 (35.3%)	11 (50.0%)	1.194	0.275
Recurrent ischeamic stroke, *n* (%)	13 (23.2%)	8 (23.5%)	5 (22.7%)	0.005	0.944
All-cause mortality, *n* (%)	23 (41.1%)	16 (47.1%)	7 (31.8%)	1.282	0.258
Stroke related mortality, *n* (%)	22 (39.3%)	16 (47.1%)	6 (27.3%)	2.192	0.139

#### Factors associated with a good outcome

To identify independent factors associated with a favorable outcome, we conducted univariable and multivariable logistic regression analyses. The univariable logistic regression analysis revealed that sex, weight, and NIHSS at two weeks were important factors related to a good prognosis. However, in the multivariable logistic regression analysis, only the NIHSS score at two weeks (OR: 0.840; 95%CI: 0.754–0.936, p = 0.002) emerged as the most significant contributor to a favorable outcome (Table 3).

**Table 3:** Analysis of independent factors associated with a good outcome

Univariate			Multivariate		
Factors	OR (95% CI)	*P*-value	Factors	OR (95% CI)	*P*-value
Sex	0.203 (0.056,0.731)	0.015*	Sex	0.197 (0.028,1.409)	0.106
Age	0.949 (0.901,1)	0.051	Weight	0.980 (0.899,1.068)	0.642
Weight	1.063 (1.005,1.125)	0.032*	NIHSS at 2 weeks*	0.840 (0.754,0.936)	0.002
STA-MCA	1.662 (0.539,5.12)	0.377			
DC	0.618 (0.202,1.889)	0.390			
Hypertension	1.173 (0.387,3.55)	0.778			
Diabetes	4.966 (0.572,43.076)	0.146			
Smoking	0.339 (0.088,1.307)	0.116			
Alcohol	0.438 (0.109,1.756)	0.244			
CHD	2.732 (0.527,14.175)	0.232			
Atrial fibrillation	4.080 (0.808,20.592)	0.089			
IV rt-PA	0.772 (0.213,2.796)	0.694			
EVT	0.438 (0.387,3.55)	0.778			
ICA	0.725 (0.225,2.334)	0.590			
MCA	1.719 (0.543,5.438)	0.357			
Initial NIHSS	1.032 (0.964,1.106)	0.366			
NIHSS at 2 weeks	0.848 (0.775,0.928)	< 0.001*			
3 month-mRS	0.180 (0.062,0.526)	0.002			

*STA-MCA* superficial temporal artery-middle cerebral artery, *IV* intravenous, *rt-PA* recombinant tissue plasminogen activator, *EVT* endovascular treatment, *CHD* Coronary heart disease, *ICA* internal carotid artery, *MCA* middle cerebral artery, *DC* decompressive craniectomy, *NIHSS* National Institutes of Health Stroke Scale, *Initial NIHSS* NIHSS at admission, *NIHSS score at 2 weeks after onset*, *mRS* modified Rankin Scale; * *p* < 0.05

#### Pre-operative perfusion analysis

**Fig. 2:**
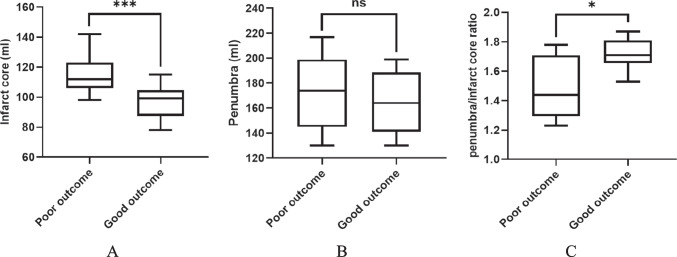
Pre-operative perfusion in patients with poor and good outcomes in the STA-MCA patients

**Fig. 3:**
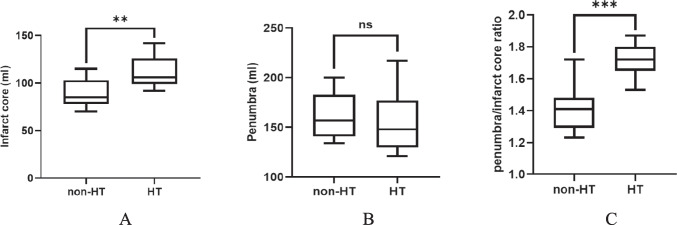
Pre-operative perfusion in patients with HT and non-HT in the STA-MCA patients

Analysis of pre-operative perfusion showed no significant differences between the STA-MCA and non-STA-MCA groups in terms of penumbra, infarct core, and penumbra/infarct core ratios. However, within the STA-MCA group, patients with poor clinical outcomes exhibited larger infarct cores (median: 112 ml versus 99 ml, p = 0.0031) and higher penumbra/infarct core ratios (median: 1.44 versus 1.71, p = 0.0133) (Fig. 2A, B, and C). Additionally, patients who experienced hemorrhagic transformation (HT) in the surgical group also had larger infarct cores (median: 85 ml versus 106 ml, p = 0.007) and higher penumbra/infarct core ratios (median: 1.41 versus 1.72, p = 0.0005) (Fig. 3A, B, and C).

### Meta-analysis

#### Study selection and quality assessment

Out of the initial 244 studies retrieved, 196 were excluded after screening titles and abstracts. The full texts of the remaining 48 studies were examined, and ultimately, seven studies met the inclusion criteria, with a total of 148 participants included in the final analysis (Supplementary Fig. 1). The mean age in these studies ranged from 50.7 years to 70.9 years (Supplementary Table 2), and the duration of follow-up ranged from 3 months to 27.5 months. All studies generally scored at least 12 points for quality, and the results of the quality evaluation are presented in Supplementary Table 2.

#### Clinical efficacy of urgent STA-MCA bypass in AIS

**Fig. 4:**
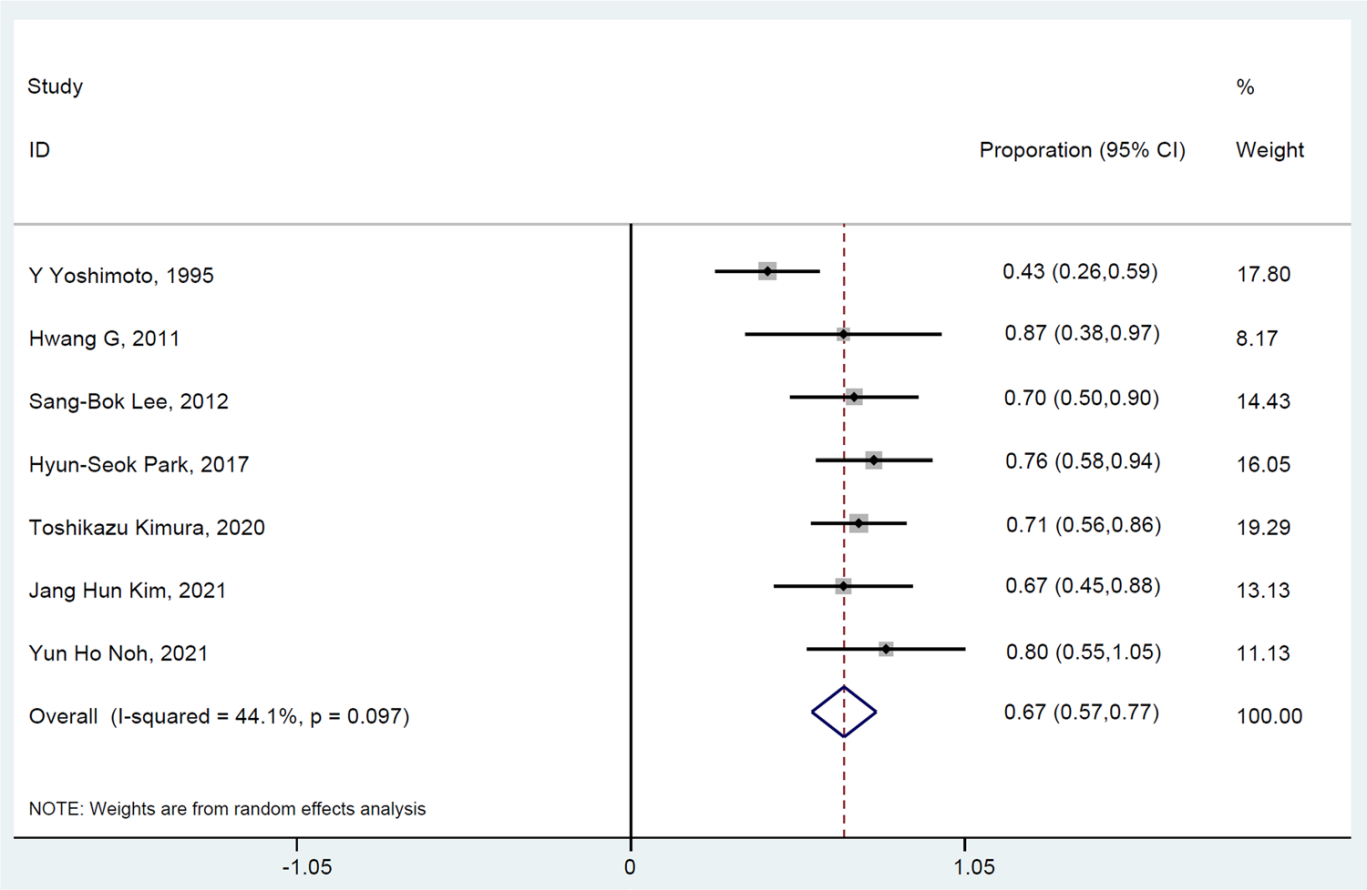
Forest plot of the good outcome incidence of urgent STA-MCA in the AIS. The squares and horizontal lines represent the incidence and 95% confidence interval (CI), respectively. The area of the squares reflects the weight of each study. The diamond represents the pooled proportion and 95% CI

**Fig. 5:**
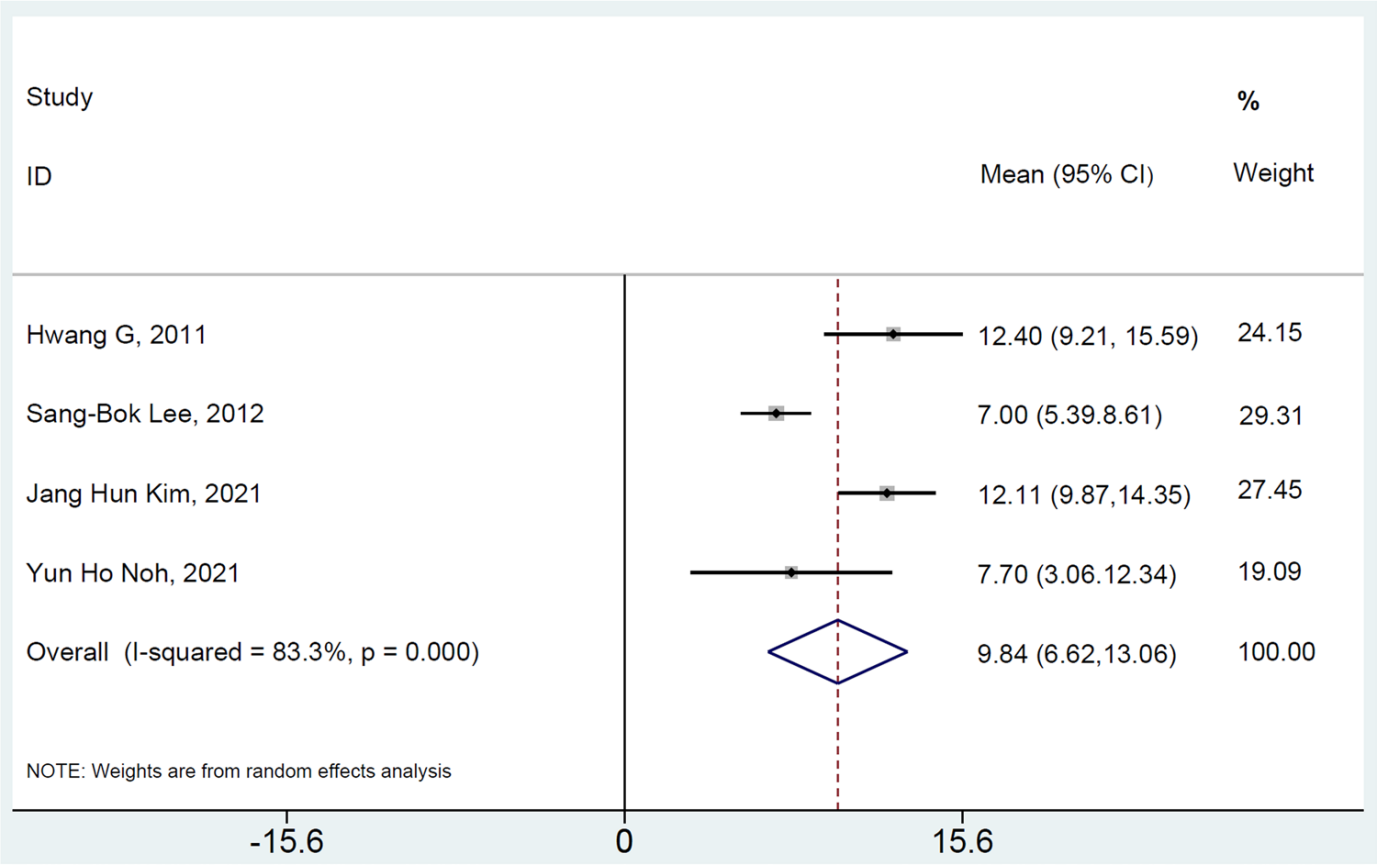
Forest plot of the mean NIHSS score before STA-MCA

A total of seven studies [15, 16, 21–25] reported the clinical efficacy of urgent STA-MCA bypass in AIS. Meta-analysis revealed that the good outcome rate for urgent STA-MCA in AIS treatment was 67% (95% CI: 57%–77%), with no significant heterogeneity (I^2^ = 44.1%, p = 0.097) across the studies, indicating that early STA-MCA bypass in AIS has a moderately high good outcome rate (Fig. 4). Four [14, 15, 21, 22] of the included studies investigated the preoperative NIHSS score (mean ± SD). Meta-analysis demonstrated that the mean preoperative NIHSS was 9.84 (95% CI: 6.62–13.06) using the random effect model due to the presence of significant heterogeneity (I^2^ = 83.3%, p < 0.001) (Fig. 5).

#### Publication bias and sensitivity analysis

No significant publication bias was found using Egger’s test (p = 0.555) (Supplementary Fig. 2). A sensitivity analysis was performed, and the results indicated that the study by Y Yoshimoto et al. [21] might be the source of heterogeneity (Supplementary Fig. 3). This particular study had a longer duration of ischemia (time from onset to surgery) compared to other studies, which may have contributed to clinical heterogeneity. Furthermore, patients in the study by Y Yoshimoto et al. underwent STA-MCA from January 1988 to May 1993, when microsurgical techniques were less advanced, possibly leading to a lower rate of good outcomes in these patients.

## Discussion

AIS-LVO, when not adequately managed in terms of hemodynamic recanalization, is associated with substantial disability and mortality [26]. STA-MCA surgery, although a relatively recent approach, has shown promise in the treatment of AIS, albeit with limited data. This led us to investigate the efficacy of early STA-MCA bypass surgery in AIS patients through a cohort study and meta-analysis.

Our meta-analysis results suggested that early STA-MCA bypass in AIS patients leads to a relatively favorable prognosis during both short-term (three months) and long-term (at least six months) follow-up periods. The studies included in the meta-analysis predominantly originated from Asian countries, including Japan and Korea. Furthermore, a substantial portion of the data from prior trials indicated that most diffusion-weighted imaging (DWI) lesions had a volume of less than 30 ml, with a mean preoperative NIHSS score of 9.84.

However, our cohort study uncovered three significant findings. Firstly, STA-MCA had a noticeable impact on early NIHSS scores, but it did not result in long-term improvements in the outcomes of AIS-LVO patients. Secondly, while STA-MCA bypass surgery did not appear to elevate the risk of hemorrhagic transformation or recurrent ischemic strokes, it did not confer a survival advantage for AIS-LVO patients. Finally, the analysis of pre-operative perfusion in our study showed that while there were no significant differences in baseline perfusion characteristics between STA-MCA and non-STA-MCA groups, within the STA-MCA group, patients with poor clinical outcomes and patients who experienced hemorrhagic transformation had larger infarct cores and higher penumbra/infarct core ratios. These findings emphasize the importance of assessing baseline perfusion characteristics in predicting outcomes following STA-MCA bypass in AIS-LVO patients and highlight the need for further research in this area.

The differences in patient selection, disease progression, and long-term complications likely contributed to the variations in outcomes between our study and the meta-analysis. First, our study specifically focused on AIS-LVO patients with high NIHSS scores before surgery. In these cases, cerebral tissue necrosis progresses gradually, increasing focal edema and intracranial pressure, thereby impacting brain perfusion. Our initial NIHSS assessments were conducted at an average of 19 hours post-onset, indicating patients were still in the early stages of cerebral edema. As edema intensified over time, patients' symptoms evolved, aligning with the natural disease progression in extensive cerebral infarction [27]. The role of STA-MCA bypass in this context is to maintain cerebral blood flow above the threshold for irreversible damage rather than immediately improving cerebral reperfusion [22]. For patients with high NIHSS scores, severe neurological deficits were already evident, and the low-flow STA-MCA bypass might not suffice to change their clinical outcome. Second, despite an improvement in postoperative hemodynamics observed in our study, STA-MCA bypass rarely prevented stroke recurrence, a significant factor contributing to poor outcomes in AIS-LVO patients [28, 29]. Moreover, many of our patients underwent EVT, and interventional vascular procedures can lead to arterial injury, causing the release of pro-inflammatory factors into the bloodstream that may enter the brain and result in secondary brain tissue injury [30]. Lastly, our study had a longer follow-up duration. The long-term complications, such as recurrent pulmonary infections, muscle stiffness, atrophy, and pressure ulcers in AIS-LVO patients, can diminish their quality of life and recovery capacity, despite the acute phase resolution of HT and cerebral edema [31].

It's important to note that DC for AIS-LVO has shown benefits in reducing mortality and improving functional outcomes [32], but our study did not support these claims. Most DC studies were conducted within 48 hours of stroke onset, which may explain this discrepancy. Age could also be a contributing factor, as research suggests that patients under 60 years old are more likely to benefit from DC [33]. Early imaging studies, like MRI and CT perfusion, can help identify which AIS-LVO patients might benefit from early DC.

While urgent STA-MCA bypass is an option for AIS patients with low NIHSS scores, it remains unclear which patient characteristics make someone a suitable candidate for the procedure and what factors contribute to good or poor outcomes. Several factors may affect the prognosis of AIS patients undergoing STA-MCA. Toshikazu Kimura et al. [25] identified sex, age, vascular lesions, initial NIHSS score, and preoperative NIHSS score as factors associated with a poor prognosis, with the preoperative NIHSS score being the most significant (OR: 1.65; 95% CI: 1.12–2.90). Consistent with prior research, our data showed the NIHSS score at two weeks after onset was a significant predictor of a poor prognosis. Therefore, patients with high NIHSS scores before surgery should undergo a careful evaluation of the benefit-risk ratio for STA-MCA treatment.

Several limitations must be acknowledged in this study. Firstly, the inclusion of studies of varying quality necessitates caution when interpreting the findings. Secondly, while Egger's test did not reveal significant publication bias in our meta-analysis, the limited number of studies may affect the test's sensitivity. Thirdly, our sensitivity analysis indicated that the study by Y. Yoshimoto et al. [21] might be a potential source of heterogeneity. In our cohort study, the analysis lacked statistical power due to the rarity of patients with large vessel occlusions undergoing surgical intervention. Additionally, the absence of randomization in the surgical and control groups introduces the potential for selection and confounding biases. Importantly, our patient selection criteria for urgent bypass were suboptimal, with many patients exhibiting large infarct cores and high penumbra/infarct ratios. This likely contributed to the poor outcomes seen, as patients are poor candidates for revascularization. Future research should focus on patients with small infarct volumes but large penumbra who are at high risk of collateral failure. Therefore, more selective inclusion criteria are warranted based on advanced imaging to identify patients likely to respond to bypass. Overall, rigorously designed randomized controlled trials with refined eligibility standards are essential to confirming the effectiveness and precise scope of this approach.

## Conclusion

While the meta-analysis suggests the potential role of STA-MCA bypass in mild to moderate AIS, our single-center study indicates that in patients with severe stroke (high NIHSS score), the STA-MCA bypass did not improve the long-term outcome.

## Supplementary Information

Below is the link to the electronic supplementary material.

Supplementary file1 (DOCX 203 KB)

## Data Availability

Data are available upon reasonable request.
